# Prevalence of Cardiometabolic Syndrome and its Association With Body Shape Index and A Body Roundness Index Among Type 2 Diabetes Mellitus Patients: A Hospital-Based Cross-Sectional Study in a Ghanaian Population

**DOI:** 10.3389/fcdhc.2021.807201

**Published:** 2022-02-09

**Authors:** Enoch Odame Anto, Joseph Frimpong, Wina Ivy Ofori Boadu, Valentine Christian Kodzo Tsatsu Tamakloe, Charity Hughes, Benjamin Acquah, Emmanuel Acheampong, Evans Adu Asamoah, Stephen Opoku, Michael Appiah, Augustine Tawiah, Max Efui Annani-Akollor, Yaw Amo Wiafe, Otchere Addai-Mensah, Christian Obirikorang

**Affiliations:** ^1^ Department of Medical Diagnostics, Faculty of Allied Health Sciences, College of Health Sciences, Kwame Nkrumah University of Science and Technology, Kumasi, Ghana; ^2^ School of Medical and Health Sciences, Edith Cowan University, Perth, WA, Australia; ^3^ Department of Molecular Medicine, School of Medicine and Dentistry, College of Health Science, Kwame Nkrumah University of Science and Technology, Kumasi, Ghana; ^4^ Department of Medical Laboratory Technology, Accra Technical University, Accra, Ghana; ^5^ Department of Obstetrics and Gynaecology, Komfo Anokye Teaching Hospital, Kumasi, Ghana

**Keywords:** cardiometabolic syndrome, type 2 diabetes mellitus, body roundness index, body shape index, anthropometric indices, risk factors, prevalence

## Abstract

Cardiometabolic syndrome (MetS) is closely linked to type 2 diabetes mellitus (T2DM) and is the leading cause of diabetes complications. Anthropometric indices could be used as a cheap approach to identify MetS among T2DM patients. We determined the prevalence of MetS and its association with sociodemographic and anthropometric indices among T2DM patients in a tertiary hospital in the Ashanti region of Ghana. A comparative cross-sectional study was conducted among 241 T2DM outpatients attending the Komfo Anokye Teaching Hospital (KATH) and the Kumasi South Hospital for routine check-up. Sociodemographic characteristics, clinicobiochemical markers, namely, systolic blood pressure (SBP), diastolic blood pressure (DBP), fasting blood glucose (FBG), and glycated hemoglobin (HbA1C) were measured. Anthropometric indices, namely, body mass index (BMI), Conicity index (CI), body adiposity index (BAI), A body shape index (ABSI), body roundness index (BRI), Waist-to-hip ratio (WHR), and Waist-to-height ratio (WHtR) were computed based on either the Height, Weight, Waist circumference (WC) or Hip circumference (HC) of the patients. Metabolic syndrome (MetS) was classified using the National Cholesterol Education Program (NCEP) Adult Treatment Panel III (ATP III) criteria. Data entry and analysis were done using Excel 2016 and SPSS version 25.0 respectively. Of the 241 T2DM patients, 99 (41.1%) were males whereas 144 (58.9%) were females. The prevalence of cardiometabolic syndrome (MetS) was 42.7% with dyslipidemia and hypertension recording a prevalence of 6.6 and 36.1%, respectively. Being a female T2DM patient [aOR = 3.02, 95%CI (1.59–5.76), *p* = 0.001] and divorced [aOR = 4.05, 95%CI (1.22–13.43), *p* = 0.022] were the independent sociodemographic predictors of MetS among T2DM patients. The 4th quartile for ABSI and 2nd to 4th quartiles for BSI were associated with MetS on univariate logistic regression (*p <*0.05). Multivariate logistic regression identified the 3rd quartile (aOR = 25.15 (2.02–313.81), *p* = 0.012) and 4th quartile (aOR = 39.00, 95%CI (2.68–568.49), *p* = 0.007) for BRI as the independent predictors of MetS among T2DM. The prevalence of cardiometabolic syndrome is high among T2DM patients and this was influenced by female gender, being divorced, and increased BRI. Integration of BRI as part of routine assessment could be used as early indicator of cardiometabolic syndrome among T2DM patients.

## Introduction

Diabetes mellitus (DM) has become a public health concern and its morbidity and mortality rates have continued to rise gradually (1). It was estimated that the global prevalence of type 2 Diabetes mellitus (T2DM) in 2019 was 7.5% (374 million) and is expected to reach 8.0% (454 million) by 2030 and 8.6% (548 million) by 2045 (2). Furthermore, reports are that in the 21st century, developing countries will face the risk of this epidemic, with 80% of all new DM cases due to occur in Sub-Saharan African countries like Ghana by 2025 (3). In Africa alone, an estimated 15.5 million adults aged 20 to 79 had diabetes, which represents a regional prevalence of 3.3% (4). Moreover, T2DM accounted for over 298,160 deaths (6% of all deaths) in Africa region that same year (4). An estimated 19.4 million adults (20–79 years) lived with diabetes in the International Diabetes Federation (IDF) Africa Region and this signifies a 3.9% regional prevalence (5). The Region with the highest fraction of undiagnosed diabetes is Africa, with 60% of adults still living with diabetes and not aware of it (5). In urban Ghana, at least 6% were diagnosed with T2DM and were related to obesity, age, and low socioeconomic status, and often leading to cardiometabolic risk factors such as hypertension and dyslipidemia (6). T2DM causes more havoc by its strong association with cardiometabolic risk factors such as dyslipidemia, metabolic syndrome and hypertension and its primary driving factor is overweight and obesity (7). In a Ghanaian population, cardiometabolic risk factors were found to have increased among urban settlers as a result of increased physical inactiveness and unhealthy eating habits among the urban settlers and the association between obesity and T2DM has been well documented (8–10). Overweight and Obesity are linked to increased cardiometabolic risk but can differ significantly depending on gender, age, eating habits, and even among subjects with morbid obesity (9).

Anthropometric indices such as BMI used to access obesity have been accepted in clinical practices due to its simplicity and usefulness for the prediction of body fat distribution in Diabetes Mellitus (10, 11). Body mass index (BMI) has been the traditional anthropometric index for general obesity diagnosis and reflects the total body fat distribution (12). BMI is however, limited by its inability to differentiate fat and muscle mass, and also overall distribution of body fat (13). A previous report has shown that the conventional anthropometric indices such as BMI could not differentiate muscle mass and body fat (14). Other anthropometric indices such as Waist Circumference (WC), Weight to Height Ratio (WHtR), Waist to Hip Ratio (WHR), Body Adiposity Index (BAI), and Conicity Index (CI) have been used to predict the various cardiometabolic risk factors in T2DM patients (15–17). Due to endpoint dissimilarity between men and women and also different racial groups, the validity of WC has also been questioned for clinical use in cardiometabolic risk assessment (13, 18). Likewise, WHR as a measure of fat distribution necessitates endpoints for ethnic group and sex (19). The use of WHtR as a standardized tool for ascertaining central obesity between varied racial groups has also been questioned (20). BAI is also limited by severe obesity and its validity has been questioned (21, 22) and optimal cut-off points of CI is also limited by sex and age (23).

The development of other anthropometric indices to improve the limitation of other anthropometric has been explored (24). Two new body indices have been formulated lately (25, 26). A new body index referred to as A Body Shape Index (ABSI) was introduced by Krakauer and Krakauer (25). This index takes into account one’s waist circumference, height, and weight. Krakauer and Krakauer discovered that ABSI values and abdominal body fat were positively correlated and recent studies have used ABSI to predict premature mortality (27, 28). Thomas et al. (26) introduced another new index called the Body Roundness Index (BRI) in 2013. This index takes WC and height into account. However, the controversy over the association of the two new body indices (ABSI and BRI) and cardiometabolic risk factors among diabetes patients is yet to be explored in a Ghanaian setting (16).

Previous studies conducted showed that the two new indices—ABSI and BRI were more related to cardiometabolic risk factors than WC and BMI (29–31). However, studies on the association of BRI and ABSI with cardiometabolic risk factors among Ghanaian Diabetes patients are non-existent. It is therefore imperative that this study is done to evaluate the relationship between the two new indices and cardiometabolic risk factors among Type 2 Diabetes patients in the Ghanaian population.

## Materials and Methods

### Study Design

A hospital-based comparative cross-sectional study was conducted between March 2021 and June 2021 at the Komfo Anokye Teaching Hospital (KATH) and Kumasi South Hospital, Agogo after obtaining permission from the Institutional Ethics Committee.

### Study Setting

KATH is a 1,200-bed facility situated in Kumasi in the Ashanti region, Ghana. The Ashanti region is situated centrally in Ghana’s middle belt and lies between longitudes 0.15W and 2.25W, and latitudes 5.50N and 7.46N. Kumasi is second only to Accra in population density. Its strategic geographic position has granted it the status of the main transport depot and guaranteed its central role in an immense and lucrative distribution of goods not only in the country but beyond. This has made KATH one of the nation’s most assessable tertiary medical centers. In Kumasi, there are nine sub-metros, including the Bantama sub-metro, where KATH is situated. KATH is Ghana’s second-largest hospital and the only tertiary health institution of the Ashanti Region. It is the primary referral hospital for Ashanti, Northern, Brong Ahafo, and Western Regions in Ghana. It also receives referrals from other neighboring countries such as Burkina Faso and Ivory Coast. For easy management and specialization, the hospital has been divided into fifteen (15) Directorates. Out of the 15, two are non-clinical and thirteen are clinical. Several clinical and non-clinical supporting units are also there. Kumasi South Hospital is situated in Agogo and is the second largest in the Southern part of Ghana. The hospital was built in 1976, as an urban health center and was later changed to be the Kumasi South Hospital. It was upgraded to the status of Ashanti Regional Hospital in 2002. The Kumasi metropolis has a total population of 3,490,030 (2021 population census).

### Study Population and Sample Size Estimation

A total of 241 T2DM patients were recruited based on the inclusion criteria of the study until the required sample size was attained. The diagnosis of type 2 diabetes mellitus was made based on the American Diabetes Association (ADA) criteria (32). The sample size was estimated using the formula n = Z^2^ × p (1 − p)/d^2^ (Charan & Biswas, 2013), where n = sample size, Z = 1.96, p = prevalence, and d = marginal error (0.05). Using a prevalence of 90.6% obtained from a similar study conducted by Agyemang-Yeboah et al. (33) in the Bantama sub-metro, the estimated sample size (n) was 124. To increase statistical power and account for non-response distribution, 241 T2DM patients were sampled for the study.

### Inclusion and Exclusion Criteria

Outpatients T2DM patients who gave consent to the study were the only ones recruited. Outpatients 30 years to 78 years who had type 2 diabetes mellitus and consented to participate were recruited and included into the study. Pregnant women and outpatients diagnosed with gestational diabetes or type 1 diabetes mellitus were excluded from the study. Individuals below 30 years were also excluded from the study and those who suffered from chronic conditions (hypertension, stroke, HIV, tuberculosis and cancer) were as well excluded from this study.

### Ethical Consideration

Approval was sought from the Committee on Human Research, Publication and Ethics (CHRPE) at the School of Medical Sciences of the Kwame Nkrumah University of Science and Technology (KNUST) and the Komfo Anokye Teaching Hospital (KATH), Kumasi and the Kumasi South Hospital. Written informed consent was sought from each participant before the commencement of the study.

### Data Collection

Participants were first educated on the purpose of the study and only those who gave consent to participate in the study were recruited. A self-reported questionnaire was used to obtain information about the name, age, gender, and sociodemographic factors such as marital status, level of education, occupation, family history of diabetes, level of physical activity, smoking status, and alcohol status of the participants.

### Blood Pressure Measurement

Blood pressure was measured by qualified nurses using a mercury sphygmomanometer and stethoscope. Recommendation of the American Heart Association (AHA) was used to take measurements from the upper left arm after participants had sat for more than 5 min (Kirkendall, Burton, Epstein, & Freis, 1967). The average value for the two measurements (with a 5-minute break interval between measurements) was recorded to the nearest 2.0 mmHg.

### Anthropometric Measurements

Anthropometric measurements included height, weight, WC, HC, BMI, WHR, WHtR, BAI, CI, and the two new indices—ABSI and BRI. The height of subjects was measured to the nearest 0.1 cm without shoes and weight was also measured to the nearest 0.1  kg with participants in light clothing. A bathroom scale (Zhongshan Camry Electronic Co. Ltd., Guangdong, China) was used to weigh the participants and their height was measured with a stadiometer (Seca 213 mobile stadiometer, Germany). During height measurement, participants stood upright with back straight, heels together, and their feet slightly apart at a 60° angle. Waist circumference (to the nearest 0.1 cm) was measured with a Gulick II spring-loaded measuring tape (Gay Mills, WI) halfway between the inferior angles of the ribs and the suprailiac crests. The hip circumference was measured at the widest diameter around the gluteal protuberance to the nearest 0.1 cm. The other anthropometric indices were calculated as follows:


WHR=waist (cm)/hip (cm) WHtR=waist (cm)/height (cm)

BMI was calculated according to Quetelet’s formula (34):


$$\text{BMI}=\frac{\text{Weight}(\text{kg})}{\text{Height}{\left(\text{m}\right)}^{2}}$$

CI was calculated from the formula (23):


$$\text{CI}=\frac{\text{Waist circumference }(\text{m})}{0.109\times \sqrt{\frac{\text{Weight }(\text{kg})}{\text{Height }(\text{m})}}}$$

The BAI was calculated from the formula (35):


$$\text{BAI}=[\text{hip circumference (cm)}÷{\text{height (m)}}^{1.5}]-18$$

ABSI was calculated from the formula (25):


$$\text{ABSI}=\frac{\text{Waist circumference }(\text{m})}{\left[{\text{BMI}}^{\frac{2}{3}}\times \text{Height }{\left(\text{m}\right)}^{\frac{1}{2}}\right]}$$

BRI was calculated by the formula (26):


$$\text{BRI}=364.2–365.5\times {(1-{\left[\text{WC/}2\pi \right]}^{2}\text{/[}0.5\times {\text{height}}^{2}\text{])}}^{\frac{1}{2}}.$$

### Blood Sampling and Biochemical Analysis

A volume of five (5) milliliters (mls) venous blood samples were collected after an overnight fast; 4 ml was dispensed into a serum separator tube and 1 ml into fluoride-oxalate tubes. After centrifugation at 500*g* for 15 min, the serum and plasma were stored at −80°C until assayed. Parameters included Fasting plasma glucose (FPG), HbA1C, total cholesterol (TC), low-density lipoprotein (LDL), triglycerides (TG), high-density lipoprotein (HDL) cholesterol, and were assayed using the COBAS INTEGRA^(R)^ 400 plus Automated Chemistry Analyzer. The protocol for the determination of the parameters was as indicated in the manufacturer’s instructions (Fortress Diagnostics Limited, Unit 2C Antrim Technology Park, Antrim BT41 1QS, United Kingdom).

### Definition of Clinicobiochemical Terms

Dyslipidemia was defined as follows: high TC (>5.0 mmol/L), high LDL-C (>3.0 mmol/L), high TG (>1.7 mmol/L), low HDL-C (<1.0 mml/L for men and <1.2 mmol/L for women) (36). Atherogenic dyslipidemia was defined as high TG levels, low HDL cholesterol levels and an increase in LDL (37). BMI was categorized into four groups according to the conventional WHO classification (38): underweight (<18.5 kg/m^2^), normal weight (18.5–24.9 kg/m^2^), overweight (25–29.9 kg/m^2^), and obese (≥30 kg/m^2^). Metabolic syndrome (MetS) was defined according to the National Cholesterol Education Program (NCEP) Adult Treatment Panel III (ATP III), without waist circumference (39); BP over 130/85 mmHg, TG >1.7 mmol/L, HDL-C levels less than 1.03 mmol/L (men) or 1.29 mmol/L (women) and fasting blood glucose over 5.5 mmol/L. Blood pressure was defined as mean SBP ≥140 mmHg and/or a mean DBP ≥ 90mmHg or previously diagnosed hypertension or patient on blood pressure lowering drugs.

### Statistical Analysis

Entry and analysis of data were done using Microsoft Excel 2016 and SPSS version 25.0. Categorical variables were presented as frequencies with percentages while continuous variables were presented as means with standard deviations or medians with interquartile ranges after checking for normality. Chi-square analysis was used to determine the association of sociodemographic characteristics such as age categories, gender, marital status, occupation and level of physical activity with MetS among the type 2 diabetes mellitus patients. Parametric data were analyzed using independent t-test whereas nonparametric data were analyzed using Mann–Whitney U-test. To assess the strength of the association between continuous variables, partial Spearman’s correlation coefficient was used. Multivariate logistic regression analysis was conducted to compare the predictive capacities of the anthropometric indices for cardiometabolic risk among the participants. P-values less than 0.05 were considered statistically significant for all analyses.

## Results

### Socio-Demographic Characteristics of the Study Population

**Table 1** shows the socio-demographic characteristics of Type 2 diabetes patients with and without metabolic syndrome. A total of 241 type 2 diabetes mellitus patients were recruited into the study of which 138 (57.3%) had no metabolic syndrome (MetS) and 103 (42.7%) had MetS.

**Table 1:** Socio-demographic characteristics of the study population.

Variables	Total(n = 241)	T2DM					
		Without MetS (n = 138)		With MetS (n = 103)		*p*-value	
**Age (years)**						0.625	
Median (IQR)	58.00 (50.00–65.00)	57.50 (50.00–66.00)		58.00 (49.00–63.00)			
**Age Categories n (%)**						0.068	
30–49	59 (24.5)	33 (23.9)		26 (25.2)			
50–59	77 (32.0)	47 (34.1)		30 (29.1)			
60–69	74 (30.7)	35 (25.4)		39 (37.9)			
70–79	31 (12.9)	23 (16.7)		8 (7.8)			
**Sex n (%)**						**<0.0001**	
Male	99 (41.1)	71 (51.4)		28 (27.2)			
Female	142 (58.9)	67 (48.6)		75 (72.8)			
**Marital status n (%)**						**0.018**	
Single	4 (1.7)	3 (2.2)		1 (1.0)			
Married	164 (68.0)	102 (73.9)		62 (60.2)			
Divorced	18 (7.5)	4 (2.9)		14 (13.6)			
Separated	7 (2.9)	3 (2.2)		4 (3.9)			
Widowed	48 (19.9)	26 (18.8)		22 (21.4)			
**Educational level n (%)**						0.765	
Tertiary	36 (14.9)	24 (17.4)		12 (11.7)			
Senior High School	57 (23.7)	33 (23.9)		24 (23.3)			
Junior High School	78(32.4)	42 (30.4)		36 (35.0)			
Lower Primary School	28(11.6)	15 (10.9)		13 (12.6)			
No former education	42 (17.4)	24 (17.4)		18 (17.5)			
**Occupation n (%)**						0.970	
Student	1 (0.4)	0 (0.0)		1 (1.0)			
Retired	32 (13.3)	19 (13.8)		13 (12.6)			
Keeping House	23 (9.5)	14 (10.1)		9 (8.7)			
Employed	152 (63.1)	87 (63.0)		65 (63.1)			
Unemployed	31 (12.9)	17 (12.3)		14 (13.6)			
Other	2 (0.8)	1 (0.7)		1 (1.0)			
**Physical activity n (%)**						0.915	
Primary sedentary	59 (24.5)	32 (23.2)		27 (26.2)			
Sedentary with frequent activity	97 (40.2)	56 (40.6)		41 (39.8)			
Primary physical	79 (32.8)	47 (34.1)		32 (31.1)			
Physical with high intensity activity	6 (2.5)	3 (2.2)		3 (2.9)			
**Family history of T2DM n (%)**						**0.006**	
Yes	184 (76.7)	99 (72.3)		85 (82.5)			
No	56 (23.3)	38 (27.7)		18 (17.5)			
**Smoking n (%)**						0.223	
Yes	33 (13.8)	22 (16.2)		11 (10.7)			
No	206 (85.2)	114 (83.8)		92 (89.3)			
**Alcohol intake n (%)**						0.991	
Yes	102 (42.7)	58 (42.6)		44 (42.7)			
No	137 (57.3)	78 (57.4)		59 (57.3)			

Data is presented as median (IQR); Mann–Whitney test or n (%); Chi-square or Fisher’s test. p <0.05 was considered significant for Type 2 Diabetes patients with and without metabolic syndrome.

n, number; IQR, Interquartile range.

Bold value indicates the statistically significant p-values.

The median age of the total participants was 58 years and statistically, there was no significant difference between the median ages of participants without MetS and those with MetS [57.50 versus 58.00, *p* = 0.625]. Majority of the participants were in the age categories 50–59 (32.0%). Age categories was however not significantly associated with MetS status of participants (*p* = 0.068). The male:female ratio of the overall participants was 1:1.43. Of the 103 participants with MetS, 72.8% were females and 28.2% were males. Gender was significantly associated with MetS status (*p <*0.0001). The highest proportion of participants was married (68.0%). Marital status of participants was significantly associated with their MetS status (*p* = 0.018). Furthermore, higher proportion of the participants had completed junior high school (32.4%), were employed (63.1%), practice sedentary lifestyle with frequent exercise (40.2%), had family history of T2DM (76.7%), were non-smokers (85.2%) and non-alcoholic beverage drinkers (57.3%). On the contrary, participants’ educational level, occupation, physical activity status, family history of T2DM, smoking status and alcohol intake status were not proportionally significantly different in terms of participants with and without MetS status (*p >*0.05).

### Clinical, Anthropometric, and Lipid Profile Variables of the Study Population

**Table 2** shows the clinical, anthropometric and lipid profile variables of the study population. Participants with MetS had significantly higher median levels of SBP (148.00 mmHg versus 132.00 mmHg, *p <*0.0001) and DBP (88.00 mmHg versus 78.00 mmHg, *p <*0.0001) compared to the participants without MetS. Levels of FBG (*p* = 0.067) and HbA1C (*p* = 0.158) were not significantly different between the two groups. Also, except for height which was significantly taller among participants without MetS than that observed for participants with MetS [1.66 m versus 1.64 m, *p* = 0.025], all the other anthropometric indices, namely, Weight, BMI, CI, BAI, ABSI, BRI, WC, HC, and WHtR were significantly higher among participants with MetS compared to those without MetS (*p <*0.05). Participants with MetS had significantly higher median concentrations of TG [1.37 mmol/L versus 1.05 mmol/L, *p <*0.0001], Coronary risk [5.40 versus 4.68, *p <*0.0001], and VLDL [0.62 mmol/L versus 0.48 mmol/L, *p <*0.0001] than that observed among participants without MetS. Conversely, HDL-C concentration was significantly lower among participants with MetS compared to participants without MetS [1.20 mmol/L versus 1.32 mmol/L, *p <*0.0001]. The median concentrations of TC and LDL-C measures were however not statistically significantly different between T2DM with and without MetS status of participants (*p* = 0.347 and *p* = 0.252 respectively).

**Table 2:** Clinical, anthropometric and lipid profile variables of the study population.

Variable	Total (n = 241)	T2DM		
		Without MetS (n = 138)	With MetS (n = 103)	*p*-value
**Clinical**				
SBP (mmHg)	136.00 (121.00–153.00)	132.00 (119.00–144.50)	148.00 (130.00–162.00)	**<0.0001**
DBP (mmHg)	81.00 (72.00–90.00)	78.00 (70.00–84.00)	88.00 (77.00–97.00)	**<0.0001**
FBS (mmol/L)	7.90 (6.30–11.40)	7.60 (5.60–11.70)	8.20 (6.98–11.00)	0.067
HbA1C (%)	8.00 (6.60–9.60)	7.80 (6.40–9.45)	8.15 (7.00–10.03)	0.158
**Anthropometrics**				
Height (m)	1.65 (1.60–1.70)	1.66 (1.62–1.71)	1.64 (1.58–1.69)	**0.025**
Weight (kg)	68.65 (60.95–80.55)	66.78 (58.98–76.36)	75.45 (65.35–84.000	**<0.0001**
BMI (kg/m^2^)	25.51 (22.57–29.30)	24.07 (21.62–27.90)	27.72 (23.74–30.86)	**<0.0001**
CI (m^3/2^/kg^1/2^)	1.31 (1.25–1.37)	1.29 (1.21–1.35)	1.34 (1.29–1.40)	**<0.0001**
BAI (%)	29.14 (24.22–33.60)	26.11 (22.61–31.33)	31.66 (27.83–35.86)	**<0.0001**
ABSI (m^11/6^kg^−2/3^)	0.083 (0.079–0.088)	0.082 (0.078–0.086)	0.084 (0.080–0.089)	**0.001**
BRI	4.70 (3.58–5.83)	3.87 (3.14–4.92)	5.18 (4.65–6.46)	**<0.0001**
WC (cm)	92.77 ± 13.05	88.10 ± 13.56	99.02 ± 9.20	**<0.0001**
HC (cm)	99.41 ± 13.60	94.83 ± 13.97	105.55 ± 10.30	**<0.0001**
WHR	0.94 ± 0.06	0.93 ± 0.07	0.94 ± 0.06	0.237
WHtR	0.56 ± 0.08	0.53 ± 0.09	0.60 ± 0.07	**<0.0001**
**Lipid profile**				
TG (mmol/L)	1.13 (0.89–1.51)	1.05 (0.84–1.35)	1.37 (0.95–1.79)	**<0.0001**
TC (mmol/L)	4.80 (3.77–5.50)	4.70 (3.80–5.40)	4.90 (93.70–5.65)	0.347
HDL-C (mmol/L)	1.30 (1.10–1.50)	1.32 (1.20–1.60)	1.20 (1.10–1.40)	**<0.0001**
LDL-C (mmol/L)	2.81 (1.93–3.61)	2.68 (1.94–3.43)	2.97 (1.93–3.78)	0.252
Coronary Risk	4.88 (3.83–5.96)	4.68 (3.58–5.53)	5.40 (4.21–6.49)	**<0.0001**
VLDL-C (mmol/L)	0.52 (0.41–0.68)	0.48 (0.38–0.62)	0.62 (0.44–0.82)	**<0.0001**

Non-parametric data is presented as median (IQR); compared using Mann–Whitney test. *p <*0.05 was considered statistically significant for Type 2 diabetes patients without metabolic syndrome versus those with metabolic syndrome. Parametric data is presented as mean ± SD; compared using independent sample t-test. *p <*0.05 was considered statistically significant for Type 2 diabetes patients without metabolic syndrome versus those with metabolic syndrome. n, number; IQR, Interquartile range; SD, Standard deviation; SBP, Systolic Blood Pressure; DBP, Diastolic Blood Pressure; FBS, Fasting Blood Sugar; HbA1C, Glycated hemoglobin; BMI, Body mass index; CI, Conicity index; BAI, Body adiposity index; ABSI, A body shape index; BRI, Body roundness index; WC, Waist circumference; HC, Hip circumference; WHR, Waist-to-hip ratio; WHtR, Waist-to-height ratio; TG, Triglycerides; TC. Total Cholesterol; HDL-C, High Density Lipoprotein Cholesterol; LDL-C, Low Density Lipoprotein Cholesterol; Coronary risk, TC/HDL-C; VLDL, Very Low-Density Lipoprotein Cholesterol.

Bold value indicates the statistically significant p-values.

### Prevalence of Cardiometabolic Risk Factors Among the Study Population Stratified by Male and Female

**Figure 1:**
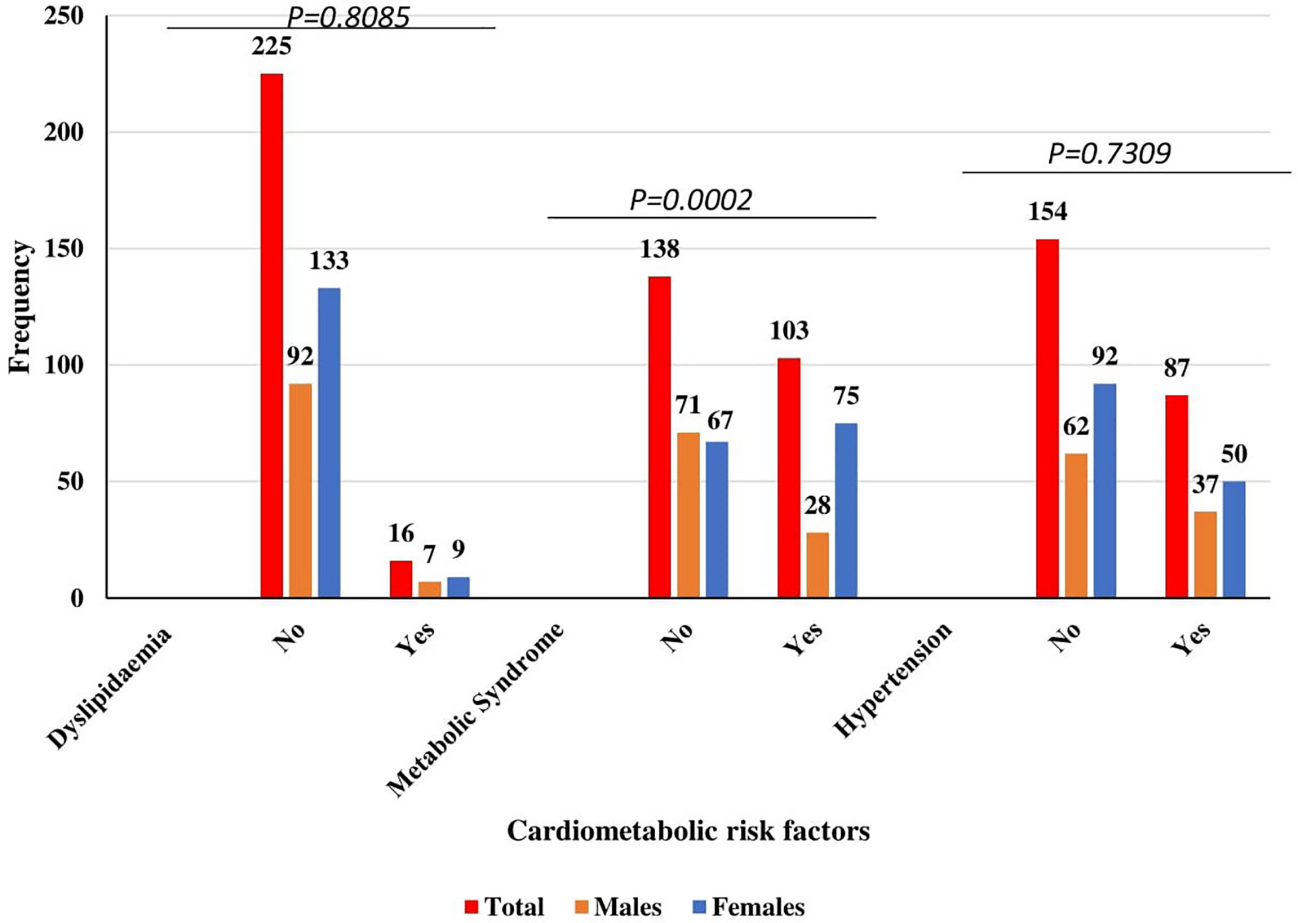
Prevalence of cardiometabolic risk factors among the study population stratified by Male and Female.

**Figure 1** shows the prevalence of cardiometabolic risk factors among the study population stratified by male and female. Of the 241 subjects, 16 had dyslipidemia, 103 had metabolic syndrome and 87 were hypertensive representing a prevalence of 6.6, 42.7, and 36.1%, respectively. Stratifying by gender, a proportion of 6.3% (9/142) of female participants had dyslipidemia, 52.8% (75/142) had MetS, and 35.2% (50/142) had hypertension. Of the 99 males, 7.1% had dyslipidemia, 28.3% had metabolic syndrome, and 37.4% were hypertensive. There was a statistically significant difference in the proportions between male and females in terms of their MetS status (*p* = 0.0002). On the contrary, there was no significant difference in the proportions between males and females in relation to dyslipidemia (*p* = 0.8085) and hypertension (*p* = 0.7309) status of the participants.

### Anthropometric Indices, Sociodemographic, Clinical indices and Lipid Measures Associated With MetS Among T2DM Patients

**Table 3** shows the odds ratios of anthropometric indices, sociodemographic, clinical indices, and lipid profile measures associated with MetS. After adjusting for possible confounders in multivariate logistic regression, BRI quartiles—Q3[a OR = 25.15, 95%CI (2.02–313.81), *p* = 0.012], Q4 [aOR = 39.00, 95%CI (2.68–568.49), *p* = 0.007], being a female [aOR = 3.02, 95%CI (1.59–5.76), *p* = 0.001] and divorced [aOR = 4.05, 95%CI (1.22–13.43), *p* = 0.022], DBP [aOR = 1.07, 95%CI (1.03–1.10), *p <*0.0001] and HDL-C [aOR = 0.10, 95%CI (0.03–0.35), *p <*0.0001] were the independent predictors of MetS among T2DM.

**Table 3:** Anthropometric indices, sociodemographic, blood pressure and lipid profile variables associated with MetS among T2DM patients.

Variables	cOR (95%CI)	*p-*value	aOR (95%CI)*	*p-*value
**ABSI quartiles**				
Q1	Ref (1)		Ref (1)	
Q2	1.98 (0.93–4.20)	0.078	1.2 (0.41–3.60)	0.734
Q3	1.12 (0.54–2.47)	0.707	0.67 (0.22–2.03)	0.482
Q4	3.56 (1.61–7.90)	**0.002**	1.80 (0.59-5.51)	0.304
**BRI quartiles**				
Q1	Ref (1)		Ref (1)	
Q2	5.15 (1.9–13.96)	**0.001**	8.04 (0.71–90.90)	0.092
Q3	14.18 (5.23–38.46)	**<0.0001**	25.15 (2.02–313.81)	**0.012**
Q4	18.78 (6.82–51.71)	**<0.0001**	39.00 (2.68–568.49)	**0.007**
**BAI quartiles**				
Q1	Ref (1)		Ref (1)	
Q2	2.85 (1.21–6.76)	**0.017**	0.50 (0.13–1.91)	0.31
Q3	7.30 (3.09–17.28)	**<0.0001**	0.65 (0.14–2.95)	0.574
Q4	7.86 (3.31–18.63)	**<0.0001**	0.33 (0.05–2.16)	0.25
**BMI categories**				
Underweight	Ref (1)		–	–
Normal weight	1.37 (0.26–7.17)	0.708	–	–
Overweight	3.32 (0.63–17.50)	0.156	–	–
Obese	5.25 (0.95–29.147)	0.058	–	–
**CI status**				
Normal	Ref (1)		Ref (1)	
High risk	8.33 (2.45–28.40)	**0.001**	3.48 (0.59–20.60)	0.17
**WHtR status**				
Normal	Ref (1)		Ref (1)	
High risk	10.38 (3.94–27.33)	**<0.0001**	0.60 (0.04–9.40)	0.717
**WHR status**				
Normal	Ref (1)		Ref (1)	
Overweight	2.41 (0.98–5.93)	0.055	1.02 (0.33–3.20)	0.971
Obese	3.80 (1.89–7.62)	**<0.0001**	0.54 (0.10–2.83)	0.462
**Sex**				
Male	Ref (1)		Ref (1)	
Female	2.84 (1.64–4.91)	**<0.0001**	3.02 (1.59–5.76)	**0.001**
**Marital status**				
Single	Ref (1)		Ref (1)	
Married	0.55 (0.06–5.39)	0.606	0.41 (0.04–4.28)	0.457
Divorced	5.76 (1.81–18.28)	**0.003**	4.05 (1.22–13.43)	**0.022**
Separated	2.19 (0.48–10.13)	0.314	1.42 (0.28–7.23)	0.676
Widowed	1.39 (0.73–2.67)	0.318	0.68 (0.31–1.53)	0.353
**SBP (mmHg)**	1.03 (1.01–1.04)	**<0.0001**	1.01 (0.99–1.03)	0.306
**DBP (mmHg)**	1.07 (1.04–1.09)	**<0.0001**	1.07 (1.03–1.10)	**<0.0001**
**TG (mmol/L)**	2.89 (1.69–4.93)	**<0.0001**	0.00 (0.00–Inf)	0.353
**TC (mmol/L)**	1.13 (0.92–1.38)	0.243	–	–
**HDL-C (mmol/L)**	0.18 (0.07–0.45)	**<0.0001**	0.10 (0.03–0.35)	**<0.0001**
**LDL-C (mmol/L)**	1.17 (0.94–1.47)	0.159	–	–
**Coronary Risk**	1.40 (1.17–1.69)	**<0.0001**	1.10 (0.86–1.40)	0.455
**VLDL (mmol/L)**	10.86 (3.32–35.53)	**<0.0001**	>100 (0.00–Inf)	0.324

Compared using univariate and multivariate logistic regression.

p <0.05 was considered significant.

*Adjusted for age and gender and marital status of participants.

cOR, Crude odds ratio; aOR: Adjusted odds ratio. Inf: infinity; Ref, reference; Q1, first quartile; Q2, Second quartile; Q3, Third quartile; Q4, Fourth quartile; ABSI, A body shape index; BRI, Body roundness index; BAI, Body adiposity index; BMI, Body mass index; CI, Conicity index; WHtR, Waist-to-height ratio; WHR, Waist-to-hip ratio; SBP, Systolic Blood Pressure; DBP, Diastolic Blood Pressure; TG, Triglycerides; TC, Total Cholesterol; HDL-C, High Density Lipoprotein Cholesterol; LDL-C, Low Density Lipoprotein Cholesterol; Coronary risk, TC/HDL-C; VLDL, Very Low-Density Lipoprotein Cholesterol.

Bold value indicates the statistically significant p-values.

### Partial Spearman Correlation Coefficients of Anthropometric Indices With Hemodynamic and Lipid Markers Among T2DM Patients

**Table 4** illustrates the partial coefficients of Spearman correlation of the anthropometric indices (ABSI, BRI, BMI, WC, BAI, CI, WHtR, and WHR) with hemodynamic indices and the lipid markers among the T2DM patients. After controlling for age and gender, the new indices—ABSI and BRI were correlated moderately (*r* = 0.406, *p <*0.0001). The BRI had a strong positive correlation with WHtR (*r* = 0.992, *p <*0.0001), WC (*r* = 0.940, *p <*0.0001), BAI (*r* = 0.895, *p <*0.0001), BMI (*r* = 0.709, *p <*0.0001), a moderate and weak correlation with CI (*r* = 0.646, *p <*0.0001), and WHR (r = 0.218, p <0.0001) respectively but showed a negative correlation with height (*r* = −0.273, *p <*0.0001). However, ABSI only showed a strong positive relationship with CI (*r* = 0.957, *p <*0.0001) but moderate correlation with WC (*r* = 0.499, *p <*0.0001), WHR (*r* = 0.461, *p <*0.0001), and BAI (*r* = 0.355, *p <*0.0001). For BMI (*r* = −0.313, *p <*0.0001), ABSI showed a moderate negative correlation. ABSI was not associated with WHR and Height of participants. Moreover, BRI showed a slight but significant positive correlation with blood pressure (DBP) and two lipid markers—TG and VLDL-C. This was however not true for ABSI as it was neither associated with blood pressure nor any of the lipid markers.

**Table 4:** Partial Spearman correlation coefficients of anthropometric measures with hemodynamic and lipid markers among T2DM patients.

	ABSI	BRI	BMI	WC	BAI	CI	WHtR	WHR
**ABSI**	1	0.406**	−0.313**	0.499**	0.355**	0.957**	0.461**	0.123
**BRI**	0.406**	1	0.709**	0.940**	0.895**	0.646**	0.992**	0.218**
**BMI**	−0.313**	0.709**	1	0.646**	0.640**	−0.030	0.677**	0.122
**WC**	0.499**	0.940**	0.646**	1	0.824**	0.725**	0.958**	0.197**
**BAI**	0.355**	0.895**	0.640**	0.824**	1	0.573**	0.908**	−0.207**
**CI**	0.957**	0.646**	−0.030	0.725**	0.573**	1	0.694**	0.162*
**WHtR**	0.461**	0.992**	0.677**	0.958**	0.908**	0.694**	1	0.188**
**WHR**	0.123	0.218**	0.122	0.197**	−0.207**	0.162*	0.188**	1
								
**Height**	0.073	−0.273**	−0.175*	0.035	−0.374**	0.022	−0.251**	−0.004
**SBP**	−0.031	0.101	**0.128**	0.105	0.063	0.013	0.104	0.105
**DBP**	0.012	0.190**	0.216**	0.216**	0.135*	0.081	0.204**	0.134
**FBS**	−0.041	−0.268**	−0.265**	−0.277**	−0.222**	−0.123	−0.265**	−0.083
**HbA1C**	−0.107	−0.259**	−0.181**	−0.245**	−0.223**	−0.169*	−0.253**	−0.079
**TG**	0.144	0.199**	0.070	0.151*	0.130	0.171*	0.190**	0.189**
**TC**	0.055	−0.054	−**0.092**	−0.054	−0.064	0.023	−0.054	0.041
**HDL-C**	−0.024	−0.270**	−0.282**	−0.275**	−0.225**	−0.110	−0.266**	−0.103
**LDL-C**	0.039	−0.034	−**0.047**	−0.021	−0.040	0.020	−0.032	0.027
**CR**	0.060	0.092	0.060	0.089	0.057	0.076	0.087	**0.094**
**VLDL-C**	0.145	0.199**	0.069	0.151*	0.130	0.172*	0.190**	0.189**

All correlation coefficients were adjusted for age and gender.

**Correlation is significant at the 0.01 level (2-tailed).

*Correlation is significant at the 0.05 level (2-tailed). The index associated with the highest correlative strength to the variable in the same row has been highlighted.

ABSI, A body shape index; BRI, Body roundness index; BMI, Body mass index; WC, Waist circumference; BAI, Body adiposity index; CI, Conicity index; WHtR, Waist-to-height ratio; WHR, Waist-to-hip ratio; SBP, Systolic Blood Pressure; DBP, Diastolic Blood Pressure; FBS, Fasting Blood Sugar; HbA1C, Glycated hemoglobin; TG, Triglycerides; TC, Total Cholesterol; HDL-C, High Density Lipoprotein Cholesterol; LDL-C, Low Density Lipoprotein Cholesterol; CR, Coronary Risk; VLDL, Very Low-Density Lipoprotein Cholesterol.

Bold value indicates the statistically significant p-values.

## Discussion

Association between type 2 diabetes mellitus (T2DM) and cardiometabolic syndrome have extensively been explored. However, for the first time, this study evaluated the prevalence of cardiometabolic syndrome and its association with two new anthropometric indices among T2DM patients in two selected hospitals in the Ashanti Region of Ghana.

The present study found 42.7% of the T2DM patients with MetS and the prevalence was higher in females than in male participants (**Figure 1**). Similarly, a cross-sectional study by Yadav et al. (40) among Indian type 2 diabetes patients reported MetS prevalence of 57.7% with females having a higher prevalence than males. The slightly higher prevalence of the previous study could be partly due to low sample size in the present study and genetic differences between Caucasians and Blacks. This study registered more females (58.9%) as compared to males (41.1%). In this present study, there was a significant association between sex of participants and their MetS status (*p <*0.0001). Furthermore, being a female was significantly associated with increased odds of having MetS as compared to being a male. This finding is consistent with previous cross-sectional studies which also reported that female type 2 diabetes patients are at higher risk of having MetS when compared to males with type 2 diabetes mellitus (T2DM) (41, 42). Less exercise, increased body weight, and an increased risk of dyslipidemia in women could be the possible reason for the higher odds of females having MetS than males (41). In this study, marital status of participants was significantly associated with MetS status. Being divorced was associated with significant 5-times increased odds of having MetS compared to being single even after possible covariates were controlled. Chung and colleagues reported a similar finding in a cross-sectional study conducted among Korean adults (43). The driving factor for this finding is however not well understood. Probable explanations could be lack of social support and living alone after being divorced which could compound the risk of having MetS (44).

Controversies still exist as to which anthropometric index best predict cardiometabolic risk among T2DM. BMI is the most widely used obesity marker and has been associated with cardiometabolic risk and type 2 diabetes (45). In this present study, the median Body Mass Index (BMI), Waist-to-Hip Ratio (WHR), Waist-to-Height Ratio (WHtR), Body Adiposity Index (BAI), and Conicity Index (CI) were significantly higher among T2DM patients who had MetS as compared to those without MetS. However, none of these anthropometric were independent predictors of MetS after multivariate logistic regression. BMI is reported to be a poor indicator of cardiometabolic syndrome compared to the other obesity indices (46–48). BMI is unable to distinguish between fat and muscle mass and also between fat compartments such as visceral adipose tissue and subcutaneous adipose tissue, which are closely linked to cardiometabolic syndrome (49). Additionally, the possible explanation for failure of BMI, BAI, CI, WC, WHR, and WHtR independently predict MetS could be due to its weaker correlation with cardiometabolic risk factors as compared to the other obesity indices as observed in this study.

In this current study, the two new indices [A Body Shape Index (ABSI) and Body Roundness Index (BRI)] were included to the traditional anthropometric indices in quest to compare their predictive capabilities for cardiometabolic risk among type 2 diabetes patients in a Ghanaian population. This current study found that ABSI could not independently predict MetS among the T2DM when it was compared to the other anthropometric indices in an adjusted multivariate logistic model. This finding is in consonant with previous studies conducted among the Caucasians (50–52). Maessen et al. (52) found that the ABSI was ineffective in identifying cardiometabolic risk factors among Netherland population. Similarly, a study conducted by Li et al. (51) showed that ABSI failed to significantly predict MetS and T2DM among overweight and obese adults The poor correlation between ABSI and MetS, on the other hand, is debatable in that previous studies have linked ABSI to some cardiometabolic risk factors (26, 53). Disparities in anthropometric measures and races may have a significant impact on the predictive value of ABSI. Although the ABSI formula was adjusted for BMI, obesity status differs for Africans, Europeans and Asians since there is differences in WC across these races (25). Furthermore, Asians are much shorter than Africans, Americans and Europeans, which may confound the predictive value of ABSI. Ethnicity has been a significant moderator in the relationship between these obesity indices and cardiometabolic risk, and it holds true for both genders. In a meta-analysis conducted by Rico-Martín et al. (54), the AUCs for all anthropometric indices in the non-Chinese population were better predictors of MetS than they were for the Chinese population. A probable reason for the failure of ABSI to superiorly predict MetS is that, it was originally formulated as a risk assessment tool to predict mortality risk in a follow-up study (25). However, we employed it in a cross-sectional study to predict MetS among T2DM patients. There is a possibility that this resulted in the failure of the ABSI to show significant predictive power compared with the other indices. Also, ABSI was moderately correlated with WC in the current study but showed negative correlation with BMI from the partial Spearman’s correlation coefficients. A negative correlation with BMI suggests an inverse relationship between the two (ABSI increases with decreasing BMI). Despite limitations of BMI and WC, increasing measures of these indices are widely standard markers to predict MetS and other cardiometabolic risk (55–58). The moderate and negative correlation of ABSI with WC and BMI respectively may have accounted for its failure to predict MetS among the T2DM. Furthermore, ABSI showed no significant correlation with the hemodynamic indices and the lipid markers from the partial Spearman’s correlation test. These markers are known predictors of MetS (59, 60) and this could be among the reasons why ABSI was ineffective to predict MetS among the subjects.

The strength of this study is that BRI but not ABSI and other anthropometric indices (BMI, WHR, WHtR, BAI, and CI) was an independent predictor of cardiometabolic risk among the type 2 diabetes mellitus patients after controlling for age, gender, and marital status of participants. BRI estimates the human as an elliptical figure and improved body fat% and Visceral Adipose Tissue (VAT)% as compared to the traditional anthropometric indices. VAT% and MetS have a well-established association (61). In the present study, BRI was the only independent predictor of MetS. This implies that only BRI was significantly associated with the higher odds of having MetS and is superior to the traditional indices in predicting MetS among the subjects. In keeping with our results, several studies have similarly reported the superior power of BRI over the traditional anthropometric indices in predicting MetS (51, 62, 63). In a meta-analysis of data pooled from more than one fifty thousand people, increased BRI odds was significantly associated with increased risk of having MetS (54). Additionally, a study conducted among obese and overweight Chinese adults found that BRI was a better predictor of MetS and T2DM (51).

The current study found that BRI had a strong positive correlation with WC and also with BMI regardless of the exclusion of BMI in the BRI formulation but a negative correlation with height after age and gender were controlled. This finding is consistent with a study by Li et al. (51). In other words, for a constant WC, height decreases whereas BMI increases and the body assumes an elliptical shape. Elliptical shape and increased odds of cardiometabolic risks has been well established (64). Also, BMI and WC have been globally accepted as a tool for predicting MetS despite some shortcomings (55–58). This may have accounted for superiority of BRI in predicting MetS owing to its strong correlation with these two markers after possible cofounders were controlled. Furthermore, partial Spearman’s correlation coefficients showed that BRI was marginal but significantly associated with blood pressure (increasing DBP) and the lipid markers (TG and VLDL-C). Several studies have linked increasing blood pressure and lipid markers to a significant increased likelihood of having MetS among T2DM (59, 60). This could possibly be another reason why BRI showed a superior predictive capacity for MetS among the subjects over the other indices.

Despite the novel findings, this study had some limitations that are worth mentioning for consideration by future studies. First of all, the female participants outnumbered the males which could have introduced a bias in the prevalence of cardiometabolic syndrome. Also, all the participants were aged (30 and above), and we could not validate that the optimal anthropometric index (BRI) would be superior to the other indices in other age groups. Sample size was also small (241) which could have introduced a bias in our analysis.

### Conclusions

The prevalence of cardiometabolic syndrome was 42.7% among the T2DM patients. Cardiometabolic syndrome was influenced by female gender, being divorced, and increased body roundness index (BRI). Integration of BRI as part of routine assessment could be used as early indicator of cardiometabolic syndrome among T2DM patients.

Further studies can be done with a larger population to establish the relationship between these two new but simple anthropometric indices and MetS among type 2 diabetes patients.

## Data Availability Statement

The raw data supporting the conclusions of this article will be made available by the authors, without undue reservation.

## Ethics Statement

The studies involving human participants were reviewed and approved by The Committee on Human Research, Publication and Ethics, Kwame Nkrumah University of Science and Technology. The patients/participants provided their written informed consent to participate in this study.

## Author Contributions

Conceptualization EOA; methodology, JF, and EOA; formal analysis, JF, EOA, and SO; investigation, JF, VCKTT, CH, and BA; original draft preparation, EOA, and JF supervision, EOA. All authors listed reviewed, edited have made a substantial, direct, and intellectual contribution to the work and approved it for publication.

## Conflict of Interest

The authors declare that the research was conducted in the absence of any commercial or financial relationships that could be construed as a potential conflict of interest.

## Publisher’s Note

All claims expressed in this article are solely those of the authors and do not necessarily represent those of their affiliated organizations, or those of the publisher, the editors and the reviewers. Any product that may be evaluated in this article, or claim that may be made by its manufacturer, is not guaranteed or endorsed by the publisher.
